# Effects of Soil Properties on K Factor in the Granite and Limestone Regions of China

**DOI:** 10.3390/ijerph17030801

**Published:** 2020-01-28

**Authors:** Man Liu, Guilin Han, Xiaoqiang Li, Shitong Zhang, Wenxiang Zhou, Qian Zhang

**Affiliations:** 1 Institute of Earth Sciences, China University of Geosciences (Beijing), Beijing 100083, China; lman@cugb.edu.cn (M.L.); xiaoqli@cugb.edu.cn (X.L.); stongzhang@cugb.edu.cn (S.Z.); zhouwenxiang@cugb.edu.cn (W.Z.); 2 School of Water Resources and Environment, China University of Geosciences (Beijing), Beijing 100083, China; zhangqian9@cugb.edu.cn

**Keywords:** soil erodibility K factor, limestone soils, lateritic red soil, red soil, yellow soil, soil profile

## Abstract

Soil erosion has become a serious ecological problem in many catchments. Soil erodibility K factor can be estimated based on a series of soil properties, however, the identification of dominant soil properties that affect K factor prediction at different soil types has been little concerned. In this study, 3 soil profiles from the Jiulongjiang River Catchment (JRC) of granite region in Fujian province and 18 soil profiles from the Chenqi Catchment (CC) of karst region in Guizhou province were selected. Soil properties, including soil particle size distribution, soil organic carbon (SOC) and soil organic nitrogen (SON) content, and soil pH, were determined, and the K factors were estimated in the erosion productivity impact calculator (EPIC) model. The soils in the granite region were characteristic for coarse texture, low SOC and SON, and strong acidity compared with limestone soils. Although the K factors in both regions ranged from 0.009 to 0.018, they were overestimated in limestone soils due to frequent soil aggregation, which enhanced soil permeability, hence reduced soil erodibility. The results of principal component analysis (PCA) and structural equation model (SEM) showed that (1) K factor estimation in the soils of the granite region mainly depended on soil texture, of which silt was the most important factor; (2) while K factor in limestone soils was mainly controlled by soil organic matter (SOM) content, other soil properties, including soil pH, clay and silt contents, could indirectly affect prediction of K factor by affecting SOM accumulation.

## 1. Introduction

Soil erosion, that is, the most general form of soil degradation, has become a global problem. Every year, about 15.2 Mg hm^−1^ soil is carried away by water and wind, of which soil erosion by water is more severe and more widespread [1]. Soil erosion causes soil productivity reduction, threatens the living environment of rural residents and society stability [2], as well as disturbs global carbon balance [1]. Sloping terrain is an important factor that affects soil erosion and can perfectly appear in a catchment scale, thus many researches about soil erosion focus on the large basin, such as the Yangtze River Basin, the Yellow River Basin, and the Pearl River Basin [3,4,5]. Soil erosion by surface runoff has been widely studied compared with soil erosion by subsurface flow because the rate of soil loss on the surface is more easily observed directly [6]. However, soil erosion by subsurface runoff also causes serious ecological problems [7], for example, serious underground soil leaks in karst region of Southwest China [6]. Undoubtedly, direct measurement of the underground soil erosion rate is hardly implemented, thus, it is necessary to estimate erodibility factor based on a series of parameters of soil properties [2,8,9].

Soil erodibility generally describes the susceptibility of soil loss to water erosion, which is affected by soil properties, including soil texture, soil organic matter (SOM) content, and soil permeability [9]. The K factor is used to quantify soil erodibility in the universal soil loss equation (USLE) and revised universal soil loss equation (RUSLE), which is defined as the amount of soil loss by runoff, rainfall, or seepage within a standard unit [8]. However, direct measurement in the field to obtain the value of the K factor is generally costly and time-consuming [4]. As a consequence, the nomographs between K factor and soil properties are frequently used to estimate the K factor [8], due to the observation of excellent correlations between K factor and soil properties [10]. Therefore, the erosion productivity impact calculator (EPIC) is widely used to estimate the K factor on the basis soil organic carbon (SOC) content and soil particle size distribution [11], and it also was applied in this study. Doubtlessly, SOC content, sand, silt, and clay percent as the parameters in the EPIC model directly affect the K factor. There are many other soil properties that can indirectly affect the K factor by affecting SOM accumulation and soil particle size distribution. For example, the organic–inorganic complexes or soil aggregates formed by the combination of SOM and soil fine particles lead to (1) reduced moving ability of fine particles [12,13], (2) increased SOM stabilization, which is benefited for SOM accumulation [14,15]; humus combines with Ca^2+^, Mg^2+^, and noncrystalline minerals, resulting in a decreasing rate of SOM decomposition [16,17,18]; soil pH, temperature, and humidity significant affect SOM decomposition rate [19]. The principal component analysis (PCA) has been widely used to identify dominant soil properties in relation to soil erodibility [20,21]. The structural equation model (SEM) has been widely used as a causal inference tool, which can quantify the relative importance of these direct and indirect factors in predicting K factor [22,23].

The Jiulongjiang River Catchment (JRC) located in Fujian province belongs to the serious red soil erosion region of southern China, which mainly results from soil nutrient depletion, understory deficiency, and concentrated erosive rainfall [24]. The Chenqi Catchment (CC) is a small karst catchment in Guizhou province, where soil erosion is characteristic for strong underground soil leak and rocky desertification [6]. Especially, soil erosion in the JRC and CC have become serious ecological problems in the last few decades, mainly resulting from excavation damage for building [25] and long-term agricultural activities [26,27,28,29], respectively. Granite and limestone rocks are widely distributed in the JRC and CC, respectively, and develop into distinct soil types that show significant differences in soil properties [26,30]. In this study, soil properties, including soil particle size distribution, SOC and soil organic nitrogen (SON) content, and soil pH, were determined in the soil profiles from the JRC and CC, and soil erodibility K factor profile distribution was estimated. The objectives of this study were (1) to assess the differences in these soil properties and K factor in the soils of the JRC and CC; (2) to identify dominant soil properties affected K factor estimation in the two regions; and (3) to quantify the relative importance of these soil properties in predicting K factor in the two regions. Soil erodibility estimations in soil profiles of the granite region and karst region were performed and the main influencing factors were determined in the present study, which would contribute to establishing some reasonable measures to control the soil erosion in both surface and subsurface.

## 2. Materials and Methods

### 2.1. Study Area

**Figure 1:**
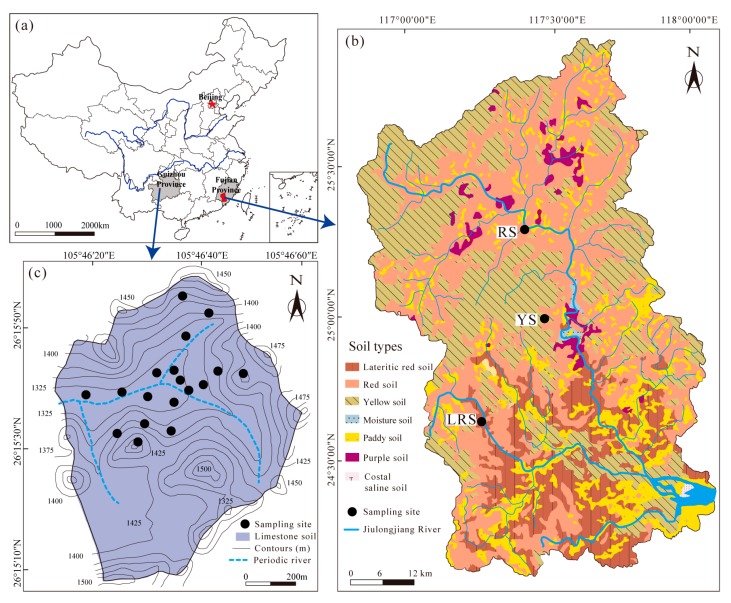
Location of study areas (**a**) and sampling sites in Jiulongjiang River Catchment (**b**) and Chenqi Catchment (**c**).

The study sites were located in Jiulong River Catchment (24°13′53″–25°53′38″ N, 116°46′55″–118°02′17″ E), Fujian province, Southeast China and Chenqi Catchment (26°15′09″–26°15′56″ N, 105°43′30″–105°44′42″ E), Guizhou province, Southwest China (Figure 1). The area of the JRC is much larger than the CC area (14,741 km^2^ vs. 1.54 km^2^). There are distinctly different elevations between them (<200 m in the JRC and 1300–1550 m in the CC, respectively). The Jiulong River is a perennial river in the JRC; while the river in the CC is a periodic river, and surface runoff occurs during the rainy season (May to October). Granite and sandstone rocks are mainly distributed in the JRC, and those develop into the lateritic red soil, red soil, and yellow soil. Limestone is dominated in the CC, which develops into limestone soil. The comparative information of climate conditions and land use between the two catchments is showed in Table 1.

### 2.2. Soil Sampling

In the JRC, three soil profiles from lateritic red soil, red soil, and yellow soil were selected, named as LRS, RS, and YS, respectively (Figure 1). In karst region, soil distribution is spatially variable with a soil thickness ranged 0.1–1.6 m [35], which is significantly lower than those in the JRC (generally >10 m) [24]. Topography conditions and land use types in the CC are more complex than those in the JRC (Figure 1 and Table 1). Thus, the more soil sites at different slope positions and under land use types can represent the whole situation of karst soils, and 18 soil profiles were selected in the CC. The depth of these profiles commonly ranged from 0.3 to 0.8 m. The interval of soil samples from profiles and total number of soil samples in the JRC and CC are introduced in Table 2.

### 2.3. Soil Analysis

Soil samples were passed through 10 mesh (2 mm) sifter after air-drying at room temperature and removing big roots and stones. Soil pH (soil:water of 1:2.5) was determined using a pH meter with a precision of ±0.05 [36]. Soil particle size distribution was measured by the laser particle size analyzer (Mastersizer 2000, Malvern, England) with a precision of ±1% [37]. The particle sizes of sand, silt, and clay were classified based on the soil texture classification systems of the United States Department of Agriculture (USDA), i.e., 0.05 mm < sand < 2 mm, 0.002 mm < silt < 0.05 mm, and clay < 0.002 mm [38].

Dried soil samples (<2 mm) were ground into powder (<150 µm) using agate mortar. Carbonates and inorganic nitrogen (NO_3_^−^ and NH_4_^+^) contained in soil samples were removed using 0.5 mol·L^−1^ hydrochloric acid (HCl) and 2.0 mol·L^−1^ potassium chloride (KCL), respectively [37]. Then these soil samples were dried at 55 °C and ground into powder for analyses of SOC and SON content in a total organic carbon analyzer (Vario TOC Cube, Elementar, Germany). Standard material of low-organic-content soil (Organic analysis standard OAS: B2152, C: 1.55 ± 0.04%; N: 0.13% ± 0.01%) was repeatedly measured to monitor the reproducibility, and the precision was better than ±0.1% for C and ±0.02% for N. The actual contents of SOC and SON should be calibrated due to the loss of carbonate and inorganic N in their removing processes [37]. The data of soil properties in the JRC and CC have been reported in [30] and [37], respectively.

### 2.4. K Factor Estimation

In the EPIC mode, soil erodibility K factor is estimated based on SOC content and the percent of sand, silt, and clay as follows [11]:

(1) $$\mathrm{Kepic}=\left\{0.2+0.3\exp\left[-0.0256S\left(1-\frac{F}{100}\right)\right]\right\}\times {\left(\frac{F}{M+F}\right)}^{0.3}\times \left[1.0-\frac{0.25C}{C+\exp(3.72-2.95C)}\right]\times \left[1.0-\frac{0.7E}{E+\exp\left(-5.51+22.9E\right)}\right]$$

where *S*, *F*, *M* (%) represent the sand, silt, and clay contents, respectively; *E* (%) = 1 − *S*/100; *C* (%) is SOC content. The *Kepic* in American unit (*Kepic*, t acre h (100·acre ft tanf in)^−1^) is translated into international unit (t ha h (ha MJ mm)^−1^) by multiplying by 0.1317.

To make the *Kepic* respect to Chinese soils, it is adjusted according to the experiential formula as follows [39]:

*K* = −0.01383 + 0.5158 *Kepic* (2)

For simplicity, the unit of K factor is omitted in the present paper. The K factor in the CC has been reported in [37].

### 2.5. Statistical Analysis

Boxplot showed the summary range of the soil particle size distribution (including sand, silt, and clay), soil pH, SOC and SON content in the soils of the JRC and the CC. The upper edge, middle horizontal line, and lower edge of box indicate 75th percentile, median value, and 25th percentile, respectively. The upper and lower whiskers indicate the maximum and minimum, respectively. The red, thick lines indicate mean values, and the blue, hollow dots indicate outliers at 5th and 95th percentiles. The Boxplots were done using SigmaPlot 12.5 (Systat Software GmbH, Erkrath, Germany).

Principal component analysis (PCA) was used to transform the original soil physiochemical properties that may affect K factor, including the percent of sand, silt and clay, soil pH, SOC and SON content, into 2 or 3 compound variables, and each compound variable was uncorrelated with all other compound variables. The principle component whose eigenvalue was exceeding 1 would be extracted, and component matrix, eigenvalues, variance, and cumulative variance were summarized. For the different soil types in the JRC and the CC, PCA was performed to determine the principle component, respectively, those soil properties contained in the first principle component were the dominant factors that affected soil erodibility in the specific catchment. The relationships between K factor and the soil properties that mainly affected it were determined by line regression analysis. Coefficient *R*^2^ and *P*-value were used to show the fitting degree of the best-fit regression line [40]. Statistical analyses, including PCA and line regression analysis, were performed using SPSS 18.0 (SPSS Inc., Chicago, IL, USA).

We used SEM to determine relative importance of soil properties (including the percent of sand, silt, and clay, soil pH, SOC and SON content) in predicting K factor at the different soil types in the JRC and the CC, and to quantify the direct and indirect effects of these soil properties. Before performing the SEM, we standardized the values of soil properties and K factor using the Z-score transformation and established a priori model based on knowledge of the direct and indirect effects on the K factor. The maximum likelihood estimation technique was used to parameterize the model with a goodness-of-fit test, including the chi-square (χ^2^), degrees of freedom (df), *p*-value, goodness-of-fit index (GFI), and the root-mean-square error of approximation (RMSEA). The *p*-value indicates the probability that the model fits the data, and a high *P*-value means the model is fitter. Other indices are also considered to indicate a good fit, e.g., χ^2^/df < 2, GFI > 0.95, RMSEA near 0 [41]. SEM were performed using the AMOS 26 (IBM, Chicago, IL, USA).

## 3. Results

### 3.1. Comparative Soil Properties in the Soils of the JRC and CC

**Figure 2:**
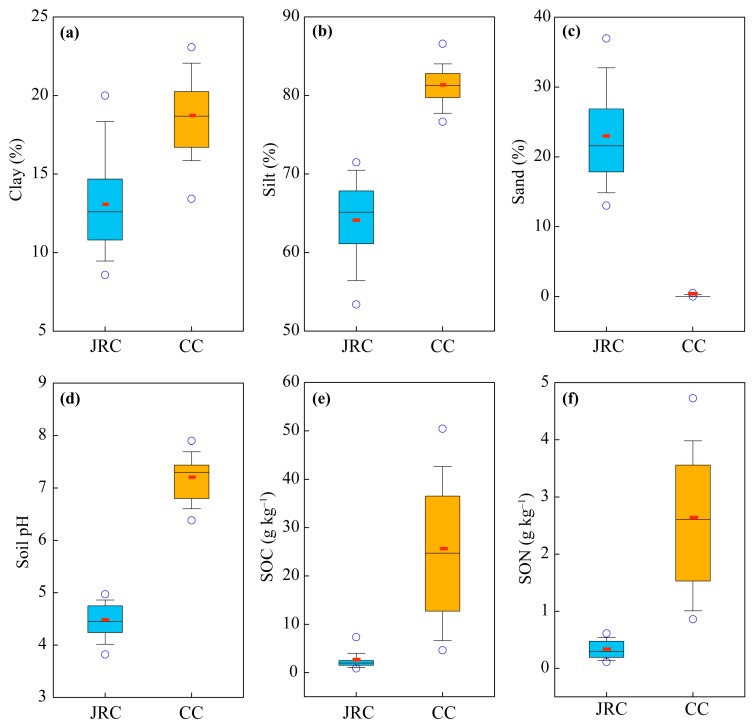
Soil properties, including (**a**) clay percent, (**b**) silt percent, (**c**) sand percent, (**d**) soil pH, (**e**) SOC content, and (**f**) SON content, in the soils of the JRC and CC. The data of the JRC and CC are cited from [30,31,32,33,34,35,36,37]. SOC, soil organic carbon; SON, soil organic nitrogen.

Clay-size particles in the soils of the JRC accounted for 9–17%, 56–71% for silt, and 15–33% for sand; while in the soils of the CC, clay accounted for 16–23%, 78–83% for silt, and nearly no sand (Figure 2a–c). Soil particle distribution in the JRC and CC fluctuated within a narrow range without obviously increasing or decreasing trend with increasing depth [30,37]. Although the soils in both the JRC and CC were classified into silt loam based on soil texture classes defined by the USDA [36], the soils in the JRC were coarser textured than those in the CC. Soil pH in the soils of the JRC ranged from 4.0 to 4.9, while it was 6.5–7.7 in the soils of the CC (Figure 2d). Soil pH in the JRC slightly decreased with increasing depth while slightly increased in the CC [30,37]. The lateritic red soils, red soils, and yellow soils were highly acid, while the limestone soils were neutral or weakly alkaline. The SOC contents in the soils of the JRC were significantly lower than those in the soils of the CC (1–4 g kg^−1^ vs. 6–44 g kg^−1^, Figure 2e), as well as the SON contents (0.1–0.5 g kg^−1^ vs. 1–4 g kg^−1^, Figure 2f). The SOC and SON contents in the JRC and CC decreased with increasing depth at the 0–50 cm depth, and almost remained constant in the soil below 50 cm [30,37]. The limestone soils contained more SOM compared with the soils in the JRC.

### 3.2. Comparative K Factor in the Soils of the JRC and CC

**Figure 3:**
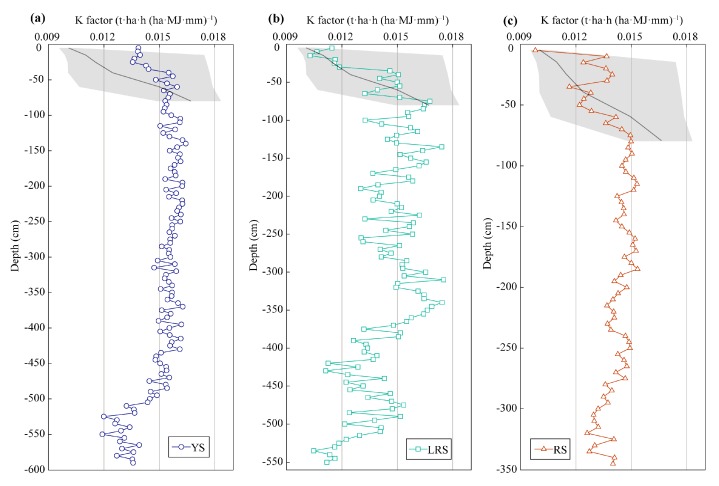
Soil erodibility K factor in soil profiles of YS (**a**), LRS (**b**), and RS (**c**) in the JRC. Black line in the 0–0.8 m layer indicates the average K factor at the same depth in the CC; the edges of shadow area are determined by maximum and minimum of K factors of them; the data are cited from [37].

Soil erodibility K factor of all soil samples in the JRC ranged from 0.0098 to 0.0175 (Figure 3), which was similar to that in the soils of the CC (0.0096–0.0184) [37]. In the 0–0.8 m layer, the K factor in the soils of both the JRC and the CC increased with increasing soil depth (Figure 3). The K factor in the LRS profile intensively fluctuated along the soil profile (Figure 3), likely associated with abrupt change in soil texture along the depth gradient. Much stable quartz was still preserved in the LRS profile after long-term chemical weathering, the different-sized quartz was unevenly distributed along the soil profile, resulting in an unstable trend in soil particle size with depth. Slight fluctuation of K factor was observed in the 0.7–4.8 m layer of YS profile and in the 0.7–2.6 m layer of RS profile (Figure 3). In the soil layer below 4.8 m of YS profile, below 2.6 m of RS profile, and below 3.8 m of LRS profile, the K factor of them decreased with increasing soil depth (Figure 3).

### 3.3. Effects of Soil Properties on K Factor in the Soils of the JRC and CC

According to the results of PCA, three principal components (PC) were extracted in the soils of both the JRC and the CC (Table 3). For soils of the JRC, The PC1 explained 41.75% of total variance and predominantly included the K factor and the percent of silt and sand; the PC2 explained 28.43% of total variance with significant loadings of SOC and SON content; and the PC3 explained 16.57% of total variance, which was mainly contributed by clay percent. While for the soils of the CC, The PC1 explained 45.06% of total variance and predominantly included the K factor and SOC and SON content; the PC2 explained 25.98% of total variance with significant loadings of the percent of clay and silt; and the PC3 explained 16.53% of total variance, which was mainly contributed by soil pH. The K factors in the soils of both the JRC and the CC were contained in the PC1, however, the first PC also associated with soil texture (silt and sand percent) or related to SOM (SOC and SON content). These results indicated that K factor in the soils of the JRC was mainly controlled by soil texture, while in the soils of the CC it depended on the abundance degree of SOM.

**Figure 4:**
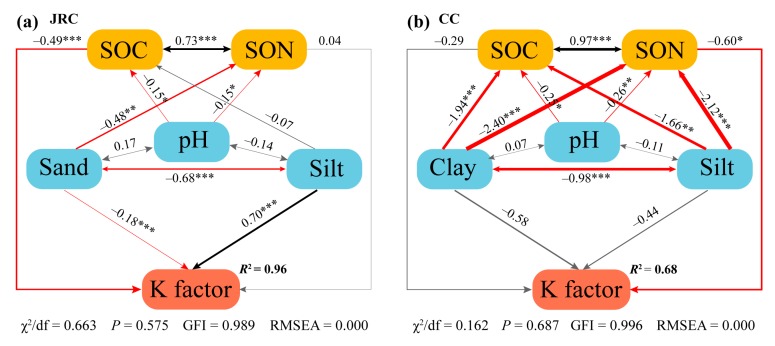
Structural equation model (SEM) evaluating the direct and indirect effects on the K factor in the JRC (**a**) and in the CC (**b**). In the SEM of the JRC, soil samples come from the profiles of YS and RS (*n* = 187, df = 3); in the SEM of the CC, soil samples come from all 18 soil profiles (*n* = 83, df = 1). Black and red lines indicate significantly positive and negative relationships, respectively; gray lines indicate the relationships are not significant at *p* = 0.05 level. The thickness of lines represents the magnitude of the path coefficient; numbers adjacent to lines are standardized path coefficients; these are analogous to relative regression weights and to indicate the effect size of the relationships. Single-headed arrows represent hypothetical causal relationships tested by the model. Double-headed arrows represent an unresolved covariance. Goodness-of-fit tests for the model are shown below the model. * *p* < 0.05, ** *p* < 0.01, *** *p* < 0.001.

**Figure 5:**
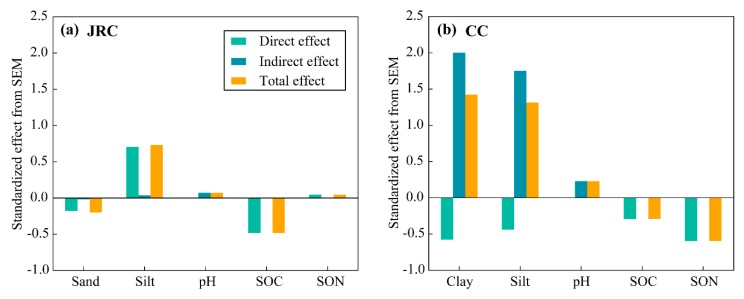
Standardized direct, indirect, and total effects derived from structural equation model (SEM) on the K factor in the JRC (**a**) and in the CC (**b**).

Soil particle size distribution in the soils of LRS profile was not accurately measured, resulting in decreasing fit effect of SEM in the JRC, thus soil samples used for SEM were selected only from the profiles of YS and RS (*n* = 187). Moreover, clay percent little affected K factor and did not have interaction with other soil properties in the SEM test of the JRC, thus clay percent was not included in the SEM. Sand percent was not included in the SEM of the CC, due to nearly no sand in this study area. According to the results of SEM, in the soils of the JRC, K factor significantly related to sand and silt percent, and SOC content (Figure 4a); while in the soils of the CC, only SON content significantly predicted K factor (Figure 4b). In the SEM of the JRC, silt percent had the greatest effect on K factor, followed by SOC content and sand percent, as indicated by the standardized direct effects in predicting the K factor (Figure 5a). According to the results of SEM in the CC, SON content had the highest direct predictive power to K factor, followed by clay percent, silt percent, and SOC content (Figure 5b). Particularly, silt and clay percent had intensive indirect effects on K factor through affecting SOC and SON content, which did not present in the JRC. Soil pH could have indirectly affected the K factor in both the JRC and the CC, however, the effect on K factor was more intensive in the CC compared with in the JRC (Figure 5).

## 4. Discussion

**Figure 6:**
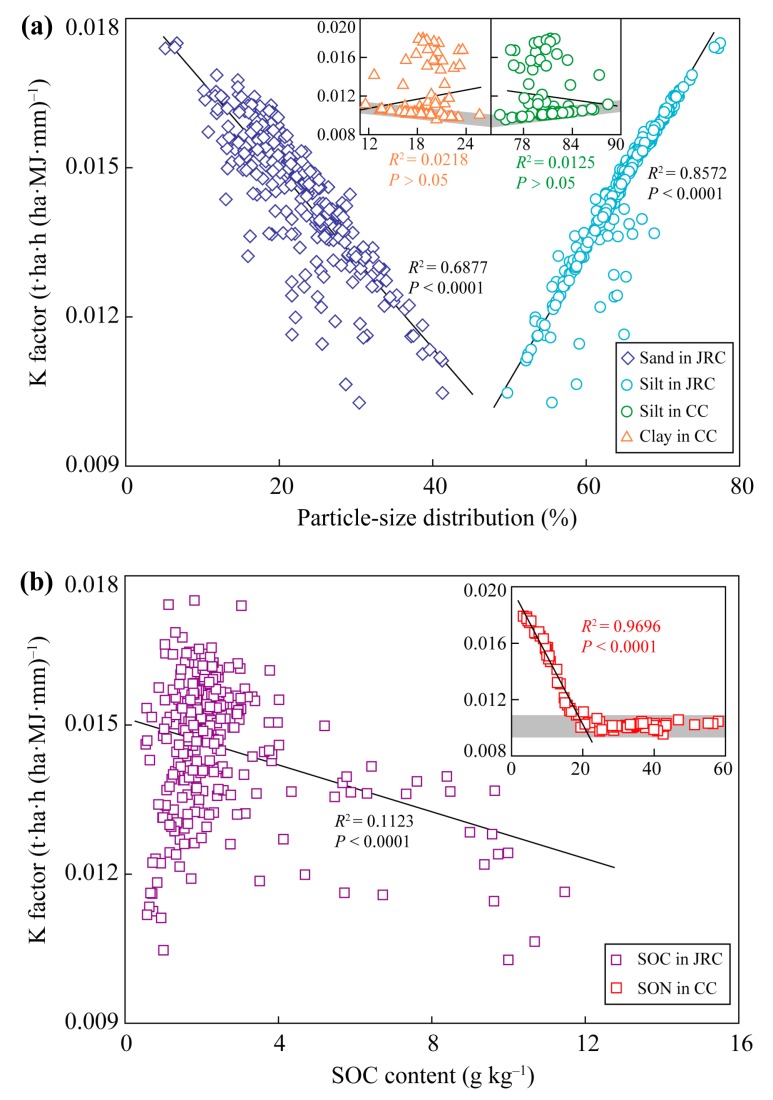
Relationships between K factor and the soil properties in the soils of the JRC and CC by line regression analysis. (**a**) Relationships between K factor and soil particle size distribution; (**b**) relationships between K factor and SOC content. The three small-sized graphs of CC were cited from an unpublished paper, the points located in the shadow area were mainly surface soil samples.

Soil properties, including soil particle size distribution, soil pH, SOC and SON content, in the soils of the JRC and CC were significantly different (Figure 2), however, the K factors in the soils of the two areas had the similar range of 0.009–0.018 (Figure 3). The K factors in the EPIC model in the JRC and CC accord with the estimated results of USLE K factor of 0.0142–0.0214 [8]. These results indicate that the soils with different soil types may have a similar degree of risk of soil erosion, thus it is necessary to identify the dominant effect factor on K factor in the different soil types. According to the results of PCA, the K factor in red and yellow soils of the JRC was mainly controlled by soil texture, while in limestone soils of the CC it was affected by SOM (Table 3). Soil particle size distribution and SOC content are the direct parameters of K factor estimation in the EPIC model [11], but why is their predictive power on K factor different in the two regions? The soils in the JRC are coarser textured and have lower SOC content than those in the CC (Figure 2). Coarse soil texture means large and more numerous soil pores, which benefits downward transport of fine particle in the process of surface water infiltration [42,43]. Soil particle organic matters easily combine with fine particles to form organic–inorganic complexes or soil aggregates, which reduces the moving ability of clay and silt [12,13]. Low SOC content in the soils of the JRC restricts the protection ability to fine particle, thus the possibility of soil erosion depends on soil particle size distribution, i.e., K factor is mainly affected by soil texture. However, in the SOC-rich and fine-textured soils of the CC, the absorption of SOM to soil water [35,44] and the immobilization for fine particles [45] effectively reduce soil erosion in the soils with low numbers of soil pores. Besides, water-stable macro-aggregates account for 30–38% of all water-stable aggregates in red soils of granite region [46], while they can achieve 63–91% in limestone soils of karst region [35]. Abundant Ca^2+^ is beneficial for soil aggregation in limestone soils, which can significantly increase soil permeability, hence reduces K factor [21]. Thus, the K factor in the CC may be overestimated. However, it is convincing that the K factor in the soils of the CC mainly depends on the abundant degree of SOM. These summaries were also verified through the significant correlations between sand (or silt) percent and K factor in the soils of the JRC, and between SOC content (when it was low than 20 g kg^−1^) and K factor in the soils of the CC (Figure 6).

Furthermore, SEM in the soils of the JRC and the CC were used to identify the direct and indirect factors and their relative importance in predicting K factor (Figure 4a and Figure 5a). According to the results of SEM, the K factor in the soils of the JRC was direct affected by SOC content, which seemed to contradict with the results of PCA. This conflict can be explained through the relationship between SOC content and K factor by line regression analysis. Significant correlation between them was observed when all soil samples were considered to participate in the analysis (SOC content: 0.5–14.4 g kg^−1^, Figure 6b). However, effective values of SOC content ranged from 1 to 4 g kg^−1^ in the boxplot (Figure 2e). Statistically, the significant correlation between them will not occur if only the effective values are considered, because the original correlation mainly depends on outliers with SOC content over 4 g kg^−1^. Thus SOC content is a spurious influence factor to K factor in the soils of the JRC. Overall, K factor in the soils of the JRC mainly depends on soil texture, of which silt percent is more important to predict it than sand percent. Similarly, there were some conflicts between the results of SEM and PCA in analyzing the effect factor to K factor in the soils of the CC, i.e., clay and silt percent could both directly and indirectly affect K factor (Figure 4b and Figure 5b). The direct effects were observed due to the significant correlation between clay (or silt) percent and K factor in the surface soils (Figure 6a). However, the K factor changed so slowly (the slope is too small) with clay and silt size distribution, thus the direct effects on the K factor were ignored (Figure 4b). Significant indirect effects of them on K factor mainly resulted from the important influences on SOM (Figure 4b), because the formations of organic–inorganic complexes or soil aggregates not only reduce the moving ability of fine particles but also increase SOM stabilization, which is beneficial for SOM accumulation [14,15]. The indirect effects of soil pH on K factor through affecting SOC and SON content in the soils of the CC were more intensive than those of the JRC (Figure 4), resulting from SOM easily accumulating in the limestone soils that have relatively high soil pH. Soil pH generally shows a nonlinear positive correlation with soil calcium carbonate (CaCO_3_) content [47], especially, in the relative high pH limestone soils of the CC, abundant Ca^2+^ would be provided. Humus produced by the decomposition of organic debris combines with Ca^2+^ to form stable complexes, which improves SOM accumulation [16,17]. K factor in the soils of the CC mainly depends on SOM content, and soil pH, clay and silt percent can indirectly affect K factor by affecting SOM accumulation. However, this way of SOM accumulation is restricted in the soils of the JRC, due to less Ca^2+^ in the soils that are developed from silicate rock. In addition, the direct effect of SOC on K factor is ignored as mentioned above, thus the indirect effect of soil pH on K factor is also not important.

The K factor in the soils of the JRC mainly depends on soil texture, which means that movement of fine particles along soil pores is the key way of soil erosion. Furthermore, the ability to reduce the movement of fine by SOM is limited in the low-organic-matter soils. Thus, the control of the soil erosion problem in the JRC needs to be considered from reducing water force on fine particles, such as increasing vegetation cover and decreasing excavation on the slope. While the K factor in the soils of the CC mainly depends on SOM content, which means that soil anti-erodibility mainly results from enhanced soil permeability and mechanical strength of soil particle through the formations of organic–inorganic complexes or soil aggregates. An advantage in the limestone soils is that the soil properties, including soil texture and soil pH, contribute to SOM accumulation. Thus, maintaining SOM storage is key to solve the soil erosion problem in the CC, such as agricultural abandonment, reducing tillage, and application of organic fertilizer.

## 5. Conclusions

The soils in the granite region of Fujian province are coarser-textured, SOC-poor, and highly acid compared with those in the limestone region of Guizhou province. Although the K factor of EPIC model in soil profiles of the two regions fluctuated in a similar range of 0.009–0.018, in limestone soils it was overestimated due to frequent soil aggregation, which enhanced soil permeability, hence reduced soil erodibility. In the granite soils, the estimation of the K factor was mainly affected by soil texture, of which silt was the most important factor. While in the limestone soils, the prediction of K factor mainly depended on SOM content; additionally, soil pH, clay- and silt-sized particles could indirectly affect the estimation of the K factor through affecting SOM accumulation. Suggestively, expansion in vegetation cover and limitation in excavation on the slope should be considered to control soil erosion in the granite region. However, abundant SOM plays a key role in maintaining soil structure in the limestone region, thus the measures that can enhance SOC storage should be preferentially considered to reduce soil erosion, for example, agricultural abandonment, reducing tillage, and application of organic fertilizer.

## Figures and Tables

**Table 1:** Comparison of Jiulongjiang River Catchment and Chenqi Catchment.

	Jiulongjiang River Catchment (JRC)	Chenqi Catchment (CC)
Latitude	24°13′53″–25°53′38″ N	26°15′09″–26°15′56″ N
Longitude	116°46′55″–118°02′17″ E	105°43′30″–105°44′42″ E
Altitude	<200 m	1300–1550 m
Area	14,741 km^2^	1.54 km^2^
Climate	Subtropical oceanic monsoon climate; MAT: 21 °C, MAP: 1400–1800 mm	Subtropical monsoonal climate; MAT: 15 °C, MAP: 1315 mm
Lithology	Granite, sandstone	Limestone
Soil type	Lateritic red soil, red soil, yellow soil, paddy soil, and purple soil	Limestone soil
Land use	Forest land: 77.8%, including coniferous forest, evergreen broad-leaf forest, and subtropical rain forest; agricultural land: 6.8%	Secondary forest land and shrub land: 45%; agricultural land: 50%

Note: MAT, mean annual temperature; MAP, mean annual precipitation. Data were cited from [30,31,32,33,34].

**Table 2:** Number of sampling profiles and total samples, profile depth, and sample interval.

	Soil Profile	Profile Depth (m)	Sample Interval (cm)	Total Sample
JRC	3	YS: 6.0; LRS: 5.5; RS: 3.5	5 cm	297
CC ^a^	18	0.3–0.8	0–30 cm: 10 cm; >30 cm: 20 cm	83

Note: ^a^ Data in the CC were cited from [35]. YS, yellow soil; LRS, lateritic red soil; RS, red soil.

**Table 3:** Principal component analysis of soil properties and K factor.

Soil Properties	Jiulongjiang River Catchment (JRC)			Chenqi Catchment (CC)		
	PC1	PC2	PC3	PC1	PC2	PC3
Clay	0.43	0.13	0.84	−0.60	0.74	−0.28
Silt	0.91	−0.06	−0.28	0.54	−0.82	0.17
Sand	−0.96	−0.03	−0.28	0.21	0.55	0.59
SOC	−0.13	0.86	0.15	0.93	0.25	−0.06
SON	0.22	0.90	−0.07	0.94	0.28	−0.01
Soil pH	−0.31	−0.57	0.47	−0.26	0.11	0.82
K factor	0.92	−0.32	−0.21	−0.81	−0.38	0.13
Eigenvalues	2.92	1.99	1.16	3.15	1.82	1.16
Variance (%)	41.75	28.43	16.57	45.06	25.98	16.53
Cumulative (%)	41.75	70.18	86.75	45.06	71.04	87.57

Note: PC, Principal component.
